# Depression leaflet translation in sindhi language

**DOI:** 10.1192/j.eurpsy.2023.1739

**Published:** 2023-07-19

**Authors:** M. I. Memon, I. Panhwar

**Affiliations:** 1 General Adult Psychiatry with Endorsement in Addiction Psychiatry, Cwm Taff University Health Board, Wales, UK, CARDIFF; 2 GP, Royal college of GP, WOKING, United Kingdom

## Abstract

**Introduction:**

We all live in a global world where all cultures have their own unique identity, a sense of belonging, and pride. In the same way I am proud to be part Sindhi culture which is originated from Indus Valley civilization. It’s a 5 thousand years old civilisation.It gave me food for thought and an opportunity to take a lead in to do a project which will not only help my college but to more than 50 million of SINDHI speaking people living across the world. As most of Siddhis live in Pakistan, India, UAE, some of them have migrated and settled in USA, Canada, UK, Australia and Ireland.

**Objectives:**

It gave me food for thought and an opportunity to take a lead in to do a project which will not only help my college but to more than 50 million of SINDHI speaking people living across the world. As most of Siddhis live in Pakistan, India, UAE, some of them have migrated and settled in USA, Canada, UK, Australia and Ireland

**Methods:**

I contacted someone in the mental health information office at Royal College of Psychiatrist and expressed my interest to help the college by contributing one more language to the college website which will be my native language SINDHI. As I wanted to translate leaflets of Royal College about various common mental health disorders in SINDHI language which would benefit more than 50 million people across the world. As they would be able to read about common mental health disorders in their own language which will help them to understand that conditions in a better way and will reduce the stigma and will encourage them to get the right help which they need and deserve.

We started working together on the project and agreed that I will do writing and he will do proof reading for it. We completed the project and handed back it over to the college representatives dealing with leaflets in other languages. After about one month’s hard work, we were lucky enough to get out a leaflet translation published by Royal College of Psychiatrist. It was a proud moment for me being a psychiatrist to help me patients and being a SINDHI to be able get my mother tongue published on Royal College website. I couldn’t have asked for more. Its not the end of this story, as we will continue to work with our college to translate more leaflets and contribute to society and to the college being a proud member of Royal College of Psychiatrist UK.

**Results:**

It was published on RCPSYCH website in translation section

I contributed one more language to RCPSYCH webiste through my work

**Conclusions:**

MY TRANSLATION WORK WAS PUBLISHED ON OFFICIAL WEBSITE OF ROYAL COLLEGE OF PSYHCIATRIST UK

IT WAS WIDLEY APPRECIATED BY WIDER COMMUNITY AND CURRENT AND EX DEAN OF ROYAL COLLEGE OF PSYCHIATRIST https://www.rcpsych.ac.uk/mental-health/translations/sindhi/depression-sindhi

**Disclosure of Interest:**

None Declared

